# Fusion of expert uncertain assessment in FMEA based on the negation of basic probability assignment and evidence distance

**DOI:** 10.1038/s41598-022-12360-9

**Published:** 2022-05-19

**Authors:** Yusong Yuan, Yongchuan Tang

**Affiliations:** 1 School of Big Data and Software Engineering, Chongqing University, Chongqing, 401331 China; 2 School of Microelectronics, Northwestern Polytechnical University, Xi’an, 710072 Shaanxi China

**Keywords:** Computer science, Electrical and electronic engineering

## Abstract

Failure mode and effects analysis (FMEA) has been widely used for potential risk modeling and management. Expert evaluation is used to model the risk priority number to determine the risk level of different failure modes. Dempster–Shafer (D–S) evidence theory is an effective method for uncertain information modeling and has been adopted to address the uncertainty in FMEA. How to deal with conflicting evidence from different experts is an open issue. At the same time, different professional backgrounds of experts may lead to different weights in modeling the evaluation. How to model the relative weight of an expert is an important problem. We propose an improved risk analysis method based on triangular fuzzy numbers, the negation of basic probability assignment (BPA) and the evidence distance in the frame of D–S evidence theory. First, we summarize and organize the expert’s risk analysis results. Then, we model the expert’s assessments based on the triangular fuzzy numbers as BPAs and calculate the negation of BPAs. Third, we model the weight of expert based on the evidence distance in the evidence theory. Finally, the Murphy’s combination rule is used to fuse the risk assessment results of different experts and calculate the new risk priority number (RPN). At the end of this paper, we apply the proposed method to analyze seventeen failure modes of aircraft turbine blades. The experimental results verify the rationality and effectiveness of this method.

## Introduction

Failure Mode and Effects Analysis (FMEA) was first proposed by NASA in 1960s. It is a tool of risk analysis and management, which aims at allocating limited resources to the projects with the highest risk^1–5^. FMEA can improve the quality, safety and reliability of the products. Due to its remarkable advantages, FMEA has been widely used in various fields, such as steel production^6,7^, fishing boat propulsion system^8^, sewage treatment^9^ and so on^10,11^. The most commonly used tool for FMEA is the risk priority number (RPN), which is expressed as the product of occurrence (O), severity (S) and detection(D). Although FMEA has significant advantages^12^, as the assessment system becomes more and more complex, the results of the assessment are subject to great uncertainty, which greatly influences the final risk analysis. Firstly, different risk factors will produce the same RPN value. For example, (O: 9, S: 2, D: 5) and (O:2, S:9, D:5), the final RPN value is same, but the occurrence and severity of the two are quite different. Secondly, traditional risk priority number treats O, S and D as equally crucial, but in fact their weights may vary. Finally, the rating of each risk factor is rated as [1, 2, 3, 4, 5, 6, 7, 8, 9, 10], so that the final RPN value will be between 1 and 1000, but not all the numbers between them can represent RPN values, in fact, there are only 120 discrete values^13,14^. When we do FMEA, we need to deal with uncertain information. At present, there are many methods to study uncertain information, such as fuzzy set theory^15–18^, D numbers theory^19^, Dempster–Shafer (D–S) evidence theory^20,21^, grey theory^22,23^, analytic hierarchy process^24,25^ and so on. Because D–S evidence theory has significant advantages in dealing with uncertain information and independent evidence fusion, Ma et al.^26^ applied D–S evidence theory to the gender analysis. In addition, Liu et al.^27^ applied D–S evidence theory to supplier selection, Perez et al.^28^ applied D–S evidence theory to 3D human motion recognition. In this study, we also choose D–S evidence theory as our research method to figure out uncertain information.

### Motivations

- In application of D–S evidence theory, how to generate basic probability assignment (BPA) is an open issue. For FMEA, we propose a novel method of generating BPAs for modeling the risk priority number in this paper.
  Quite a few scholars have put forward their own solutions to this problem. Jiang et al. proposed using fuzzy set theory to solve evidence conflict^29^, Su et al. used Gaussian function to construct BPAs^30^. Tang et al. used triangular distribution to construct BPAs for incomplete and uncertain information^31^. Mendonca et al. used game theory to solve data conflicts^32^. The base BPAs is an option in generating BPAs considering potential conflict information^33^. In this paper, we firstly construct BPAs through triangular fuzzy numbers to reduce the evidence conflict, and then consult the negation^34,35^ of BPAs to study the uncertainty of uncertain information from another angle.
- Due to the different professional backgrounds of different experts, the assessment weight of each risk factor for the same failure mode may be different.
  As an important reference in the field, expert knowledge plays a vital role in FMEA^36^. However, the evaluation of each failure mode by experts is based on their own subjective evaluation, which aggravates the uncertainty and ambiguity of the evaluation results^37^. Based on this consideration, we calculate expert weight by the evidence distance^38,39^, and finally fuse the evidence with expert weight. Other solutions for conflict data fusion can be belief entropy-based methods^40,41^.

### Contributions

Compared with a large number of FMEA methods, the main contributions of the method proposed in this paper are as follows:

- To reduce the evidence conflict, we introduce triangular fuzzy numbers to construct BPAs. In fact, different scholars have proposed many methods to reduce the evidence conflict. Bi et al.^42^ proposed a method to reduce the evidence conflict based on Tanimoto measure. Miao et al.^43^ achieved the goal of reducing the evidence conflict by modifying the D–S combination rule. Hu et al.^44^ used feature fusion method to solve this problem. Generally speaking, there are two main methods to reduce evidence conflict. One is to modify the preliminary test information, and the other is to modify the combination rule. Our research integrates these two methods. And then the idea of construct the negation^45,46^ is firstly applied to the failure mode and effects analysis.
- The evidence distance is used to calculate the weight of the experts participating in the risk assessment, which reduces the uncertainty and ambiguity of the assessment results caused by the subjectivity of the expert assessment. As a matter of fact, there are many ways to get the weight of experts, including Deng entropy^47^, AMWRPN^48^, Ambiguity Measure^49^ and so on^50–52^. These methods show good characteristics in some attributes. Compared with these methods, the evidence distance can accurately calculate the distance between two bodies of evidence, so as to provide a reliable guarantee for calculating similarity, which is why we choose evidence distance to calculate expert weight.

### Organization

In this paper, the rest of the content is arranged as follow. In “Basic concepts” section, we briefly reviewed the basic concepts. “Improved method based on the negation of basic probability assignment and the evidence distance” section  proposed the new FMEA method. “Application” section  provided the application of the proposed method. “Discussion” section  discussed the experimental results. “Conclusions” section  made the conclusion of the whole paper.

## Basic concepts

In this part, we introduce some basic concepts, including D–S evidence theory, the negation of BPA, Murphy combination rule, the evidence distance, triangular fuzzy numbers and risk priority number(RPN).

### Dempster–Shafer evidence theory^20,21^

#### Definition 1

Frame of discernment $\Theta$ is defined as a non-empty set $\Theta =\left\{ \theta _{1}, \theta _{2}, \ldots , \theta _{i}, \ldots , \theta _{m}\right\}$ , it contains *m* mutually exclusive events. The power set $\theta$ of the frame of discernment contains $2^{m}$ elements, it is shown as follows:

1 $$\begin{aligned} 2^{\Theta }=\left\{ \varnothing , \left\{ \theta _{1}\right\} , \left\{ \theta _{2}\right\} , \ldots \left\{ \theta _{m}\right\} , \left\{ \theta _{1}, \theta _{2}\right\} , \ldots , \theta \right\} \end{aligned}$$

#### Definition 2

Within the frame of discernment, the basic evidence function is defined to represent uncertain information, *mass* function *m* is the mapping of set $2^\Theta$ on [0, 1], the mapping satisfies the following relationship:

2 $$\begin{aligned}&m(\varnothing )=0 \end{aligned}$$

3 $$\begin{aligned}&\quad \sum _{A \in \Theta } m(A)=1 \end{aligned}$$

*m*(*A*) is the mass function value of proposition subset A, also known as Basic Probability Assignment (BPA), the sum of all *mass* functions is 1. We call A fatal element if *m*(*A*)>0.

#### **Definition 3**

Under the framework of evidence theory, two groups of independent *mass* functions $m_{1},m_{2}$ can be fused by the following Dempster’s combination rule^53,54^:

4 $$\begin{aligned}&m(C) = \left( {{m_1} \oplus {m_2}} \right) (C) = \frac{{\sum \limits _{X \cap Y = C} {{m_1}} (X) \times {m_2}(Y)}}{{1 - k}} \end{aligned}$$

5 $$\begin{aligned}&k = \sum \limits _{X \cap Y = \emptyset } {{m_1}} (X) \times {m_2}(Y) \end{aligned}$$

We call *k* the conflict coefficient.

#### **Definition 4**

For *n* groups of independent *mass* functions $m_{1},m_{2}...m_{n}(n>2)$, Murphy calculates the average of *n* groups of *mass* as $m_{avg}$, then iterate (*n*-1) times to get the new *mass* function. Murphy combination rule is defined as follows:

6 $$\begin{aligned}&{m^1} = {F_{DS}}({m_{avg}}, {m_{avg}}) \end{aligned}$$

7 $$\begin{aligned}&{m^i} = {F_{DS}}({m^{i - 1}}, {m_{avg}}) ({i \ge 2}) \end{aligned}$$

where $F_{DS}$ represents the Dempster’s combination rule.

### The negation of BPAs^55,56^

#### **Definition 5**

In the evidence theory,Yagar proposed a concept of negation for probability distribution. The main ideas are as follows:

8 $$\begin{aligned} {{\bar{m}}_i} = \frac{{1 - {m_i}}}{{n - 1}} \end{aligned}$$

where *n* is the amount of fatal element, and ${{\bar{m}}_{i}}$ satisfies:

9 $$\begin{aligned}&{{\bar{m}}_{i} \in [0,1]} \end{aligned}$$

10 $$\begin{aligned}&{\sum \limits _{i = 1}^n {{{{\bar{m}}}_i}} = \frac{1}{{n - 1}}\sum \limits _{i = 1}^n {(1 - {m_i}) = 1} .} \end{aligned}$$

### Evidence distance^57^

#### **Definition 6**

In order to measure the similarity between evidences, we use the distance function. In the frame of discernment $\Theta$ where has *N* elements, the evidence distance between two bodies of evidence($m_{1}$,$m_{2}$) can be defined as:

11 $$\begin{aligned} {d}({m_1},{m_2}) = \sqrt{\frac{1}{2}{{(\overrightarrow{{m_1}} - \overrightarrow{{m_2}} )}^{\text {T}}}{{\underline{D}}} (\overrightarrow{{m_1}} - \overrightarrow{{m_2}} )} \end{aligned}$$

where ${\underline{D}}$ is a matrix that has ${2^N}$ rows and ${2^N}$ columns. The elements in the matrix are:

12 $$\begin{aligned} D(A,B) = \frac{{|A \cap B|}}{{|A \cup B|}}\;\;\;\;\;A,B \in m(\Theta ). \end{aligned}$$

### Triangular fuzzy numbers^58,59^

In the fuzzy set theory, the probability that the element *x* belongs to a set *A* can be represented by a value $f_{A}(x)$ in the interval [0, 1], the membership function $f_{A}(x)\in [0,1]$.

#### **Definition 7**

Let *X* be the domain of discourse, set $A=\left\{ \left( x, f_{A}(x) \mid x \in X\right\} \right.$, the generalized fuzzy number is a fuzzy set defined on the real number, it can be expressed as ${\tilde{A}}=\left( a_{1}, a_{2}, a_{3}, a_{4} ; \omega \right)$ , of which $0 \le \omega \le 1$ , $a_{1}, a_{2}, a_{3}, a_{4}$ are real numbers. If the membership function of fuzzy number ${\tilde{A}}$ can be expressed as

13 $$\begin{aligned} {\mu _{{\tilde{A}}}}(x) = \left\{ {\begin{array}{*{20}{c}} {\frac{{\omega \left( {x - {a_1}} \right) }}{{{a_2} - {a_1}}}}&{}\quad {0 \le {a_1}< {a_2}}\\ \omega &{}\quad {x = {a_2} = {a_3}}\\ {\frac{{\omega \left( {{a_4} - x} \right) }}{{{a_4} - {a_3}}}}&{}\quad {{a_3} < x \le {a_4}}\\ 0&{}\quad {\mathrm{{ others }}} \end{array}} \right. \end{aligned}$$

then, fuzzy numbers ${\tilde{A}}$ is called triangular fuzzy numbers.

### Risk priority number (RPN)

FMEA method is used for risk analysis based on the RPN number. RPN is expressed as the product of three risk factors: occurrence (*O*), severity (*S*) and detection (*D*).

#### **Definition 8**

RPN is defined as follows:

14 $$\begin{aligned} RPN=O \times S \times D. \end{aligned}$$

Generally speaking, for each failure mode item, the risk level of each risk factor is divided into ten grades. The rating standard of occurrence, severity and detection can be found in^60^.

## Improved method based on the negation of basic probability assignment and the evidence distance

**Figure 1:**
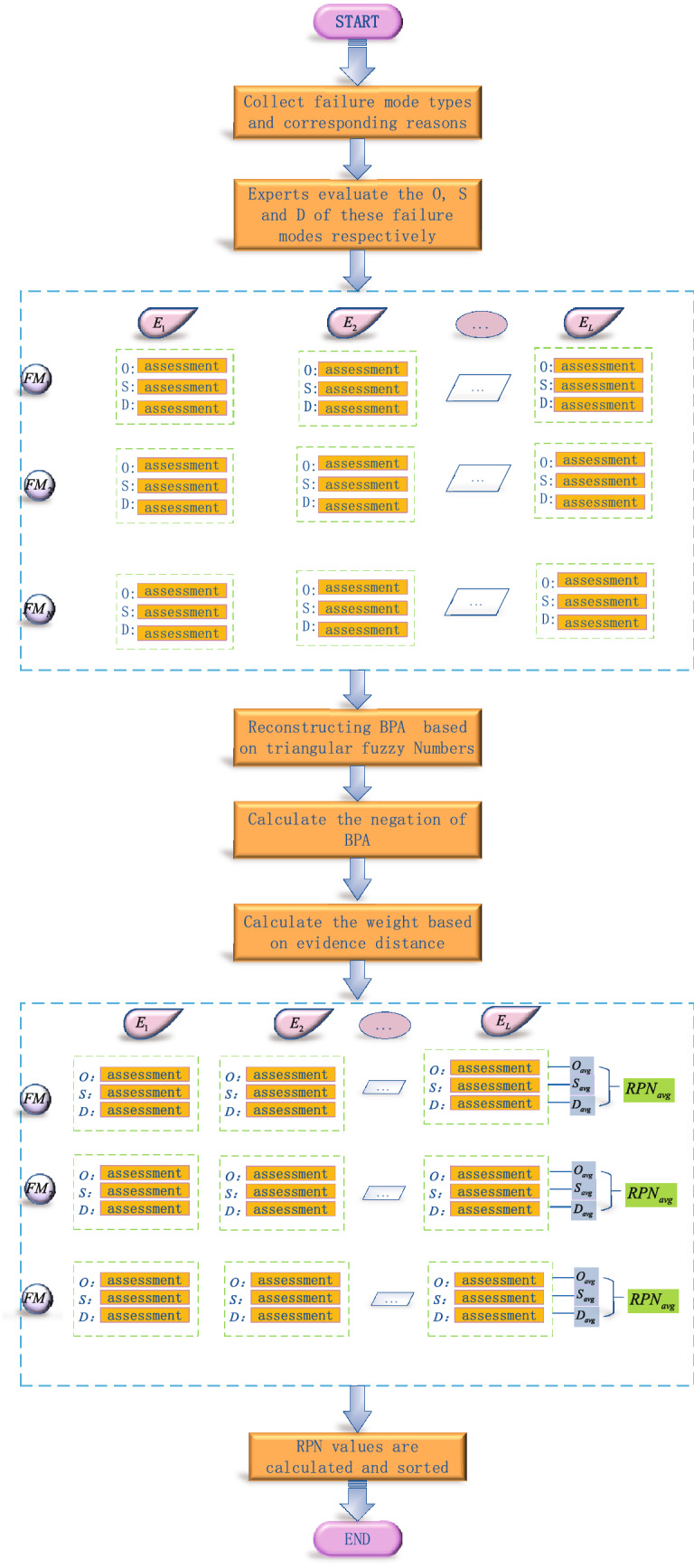
Flow chart of improved FMEA method based on triangular fuzzy numbers, the negation of BPAs and the evidence distance.

The flow chart of the improved FMEA method based on triangular fuzzy numbers, the negation of BPAs and the evidence distance in D–S evidence theory is shown in Fig. 1.

*Step 1: Simplify the frame of discernment. * Suppose there are *J* experts in an FMEA, and *N* failure modes: $E_{1}, \ldots E_{J}; FM_{1}, \ldots FM_{N}$. In this case, the frame of discernment of the *i*th risk factor of the *n*th failure mode can be written as follows:

15 $$\begin{aligned} \Theta _{i}^{n}=(1, 2, 3 ,4, 5, 6, 7, 8, 9, 10) \quad i=O, S, D ; n=1, 2, \ldots N \end{aligned}$$

Obviously, we can observe that the number of recognition frames is 3*N*. Because the evaluation of the *i*th risk factor by different experts on the *n*th failure mode is not very different, in practical applications, the frame of discernment can be simplified into the following form:

16 $$\begin{aligned} \Theta _{i}^{n}=\left( \left. \min X\right| _{X \subseteq \Theta _{i}^{n}},\left. \min X\right| _{X \subseteq \Theta _{i}^{n}}+1,\left. \ldots \max X\right| _{X \subseteq \Theta _{i}^{n}}\right) \end{aligned}$$

Among them, $\left. \min X\right| _{X \subseteq \Theta _{i}^{n}}$ represents the lowest level of evaluation by *L* experts on the *i*th risk factor of the *n*th failure mode. And, the following constraints are also satisfied:

17 $$\begin{aligned} 1 \le \left. \min X\right| _{X \subseteq \Theta _{i}^{n}} \le \left. \max X\right| _{X \subseteq \Theta _{i}^{n}} \le 10 \end{aligned}$$

**Figure 2:**
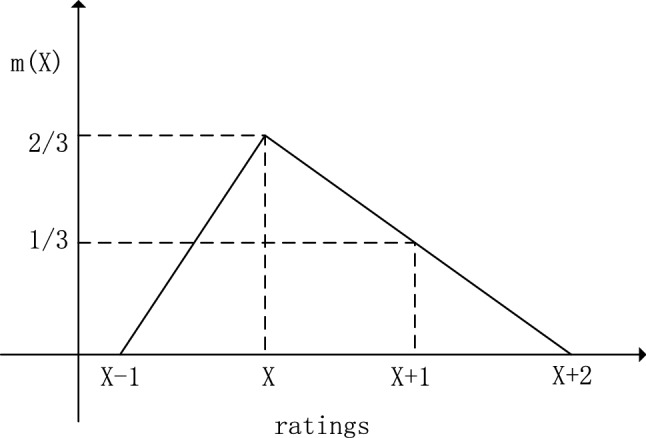
Construct new BPAs with triangular fuzzy numbers.

*Step 2: Construct BPAs using triangular fuzzy numbers. * In this step, in order to solve the conflict of combined evidence, we use triangular fuzzy numbers to construct more flexible BPAs, and fully consider the uncertainty of experts in evaluation. Since the evaluation of risk factor *i* by different experts on the *n*th failure mode is not very different, we can select two adjacent setting values to construct the BPAs function. An illustration of constructing new BPAs with triangular fuzzy numbers is shown in Fig. 2.

Based on the above discussion, we construct a trigonometric fuzzy number that fits this description well. In this example, we define $\omega =2 / 3, a_{2}- a_{1}= 1, a_{4}- a_{3}= 2$. There are two reasons: (1) The triangular fuzzy numbers cover the two adjacent tuning values, and the corresponding function value of the other tuning values is 0. (2) The sum of these two adjacent setting values is 1, which conforms to the basic definition of *mass* function.

Suppose we use *X* to represent the single possible rating of the *i*th risk coefficient of the *n*th failure mode, the new BPAs will be expressed in the following form:

18 $$\begin{aligned} & m(X)=2 / 3, \\ & m(X+1)=1 / 3 \end{aligned} $$

where the frame of discernment can be expressed as $\left[ \left. \min X\right| _{X \subseteq \theta _{i}^{n}},\left. \max X\right| _{X \subseteq \theta _{i}^{n}}+1\right]$.

### *Example 1*

Suppose that the severity(*S*) results of two experts($E_1, E_2$) for *n*th failure mode are $m_{S1}^{n}=1$ and $m_{S2}^{n}(4)=1$ respectively, using Eq. (5) we can get the conflict coefficient $k=1$. But judging from our experience, level 3 and level 4 are not completely in conflict. Then using triangular fuzzy numbers we can get new BPAs:

$$\begin{aligned} \begin{array}{l} {E_1}:m_{S1}^{n}(3) = 0.667\;\;\;m_{S1}^{n}(4) = 0.333\\ {E_2}:m_{S2}^{n}(4) = 0.667\;\;\;m_{S2}^{n}(5) = 0.333 \end{array} \end{aligned}$$

Using Eq. (5) again, we can get $k=0.78$. Obviously, the use of triangular fuzzy numbers can effectively reduce the evidence conflict.

*Step 3: Calculate the negation of BPAs. * In order to study the probability distribution from another point of view, we use the method of finding the negation of mass function. For these new BPAs that constructed with triangular fuzzy numbers, using Eq. (8) to calculate the negation of them.

*Step 4: Calculate the evidence distance. * Murphy combination rule is just a simple averaging process for BPAs, the difference between information is not considered. In our modified combination rule, we use the evidence distance to calculate weight when combining different evidences. In this step, we need to get the evidence distance between experts. Then, use these distances, we construct a distance matrix. For 3 experts, the distance matrix of *i*th risk factor and *n*th failure mode can be defined as follows:

19 $$\begin{aligned} {d} = \left( {\begin{array}{*{20}{c}} {{d}({m_{i1}^{n}}, {m_{i1}^{n}})}&{}{{d}({m_{i1}^{n}}, {m_{i2}^{n}})}&{}{{d}({m_{i1}^{n}}, {m_{i3}^{n}})}\\ {{d}({m_{i2}^{n}}, {m_{i1}^{n}})}&{}{{d}({m_{i2}^{n}}, {m_{i2}^{n}})}&{}{{d}({m_{i2}^{n}}, {m_{i3}^{n}})}\\ {{d}({m_{i3}^{n}}, {m_{i1}^{n}})}&{}{{d}({m_{i3}^{n}}, {m_{i2}^{n}})}&{}{{d}({m_{i3}^{n}}, {m_{i3}^{n}})} \end{array}} \right) \end{aligned}$$

*Step 5: Find out the support degree and credibility between the evidences. *

### **Definition 9**

Because we have performed the inverse operation on BPAs, the greater the evidence distance, the smaller the similarity. Similarity represents the degree of similarity between two bodies of evidence, the similarity matrix is defined as follows:

20 $$\begin{aligned} Sim = \left( {\begin{array}{*{20}{c}} {1 - d(m_{i1}^n,m_{i1}^n)}&{}{1 - d(m_{i1}^n,m_{i2}^n)}&{}{1 - d(m_{i1}^n,m_{i3}^n)}\\ {1 - d(m_{i2}^n,m_{i1}^n)}&{}{1 - d(m_{i2}^n,m_{i2}^n)}&{}{1 - d(m_{i2}^n,m_{i3}^n)}\\ {1 - d(m_{i3}^n,m_{i1}^n)}&{}{1 - d(m_{i3}^n,m_{i2}^n)}&{}{1 - d(m_{i3}^n,m_{i3}^n)} \end{array}} \right) \end{aligned}$$

### **Definition 10**

The degree of support reflects the extent to which a certain body of evidence is supported by other bodies of evidence. The following equtaion shows the extent to which the *i*th body of evidence is supported by other bodies of evidence.

21 $$\begin{aligned} Sup({m_i}) = \sum \limits _{\begin{array}{*{20}{c}} {j = 1}\\ {j \ne i} \end{array}}^n {Sim({m_i},{m_j})} \end{aligned}$$

In the end, we normalize the degree of support and call it credibility.

### **Definition 11**

The purpose of normalization is to make the final result more accurate. The calculation method is as follows:

22 $$\begin{aligned} Crd({m_i}) = \frac{{Sup({m_i})}}{{\sum \nolimits _{i = 1}^n {Sup({m_i})} }} \end{aligned}$$

*Step 6: Use the modified combination rule to fuse the evidence. * Using credibility as weight to average BPAs. For proposition subset A, we can get

23 $$\begin{aligned} m_i^n(A) = Crd(m_{i1}^n) \times m_{i1}^n(A) + Crd(m_{i2}^n) \times m_{i2}^n(A) + Crd(m_{i3}^n) \times m_{i3}^n(A)\quad i = O,S,D \end{aligned}$$

where *A* represents the rating standard and the value range of *A* is from 1 to 10. Then, after two rounds of iterations using Murphy combination rules, we can get the fused BPA value(${\tilde{m}}_i^n(A)$).

*Step 7: Get the new RPN value based on the improved method.* RPN is a discrete random variable. In the *n*th failure mode, it is assumed that RPN has several different levels, each of which corresponds to different probabilities. The mean value of RPN can be used to compare the overall risk of each failure mode. The specific definition is as follows:

24 $$\begin{aligned}&i_{avg}^n = \sum \limits _{A = 1}^{10} A \times {\tilde{m}}_i^n(A){} {} {} {} {} {} {} {} {} {} i = O, S, D; n = 1, 2, \ldots , N{} \end{aligned}$$

25 $$\begin{aligned}&RPN_{avg}^n = O_{avg}^n \times S_{avg}^n \times D_{avg}^n \end{aligned}$$

## Application

This section adopts a structure similar to literature^60^.

In the FMEA for aircraft compressor rotor blades, according to literature^60^, the evaluation results of the O risk factors of the first failure mode by three experts are as follows: $O_{1}(3, 40\%; 4, 60\%), O_{2}(3, 90\%; 4, 10\%), O_{3}(3, 80\%; 4, 20\%)$. This means that “the operation of the system can continue, but the performance of the system or product will be affected.”

Firstly, for the risk factor *O* of the first failure mode in^60^, the frame of discernment of risk level can be simplified as:

$$\begin{aligned} \Theta _{O}^{1}=(3, 4) \end{aligned}$$

According to Eq. (4), the new BPAs constructed from the data in literature^60^ is shown in Table 1. As for the risk factor O of the first failure mode, the results are shown in Table 2. By using Eq. (8), we can get the negation of the BPAs. The results are shown in Table 3.

**Table 1:** BPAs constructed using triangular fuzzy numbers.

Item	Rating of risk factor								
	Expert 1			Expert 2			Expert 3		
	O	S	D	O	S	D	O	S	D
1	m(3) = 0.4	m(7) = 0.667	m(2) = 0.667	m(3) = 0.9	m(7) = 0.667	m(2) = 0.667	m(3) = 0.8	m(7) = 0.667	m(2) = 0.667
	m(4) = 0.6	m(8) = 0.333	m(3) = 0.333	m(4) = 0.1	m(8) = 0.333	m(3) = 0.333	m(4) = 0.2	m(8) = 0.333	m(3) = 0.333
2	m(2) = 0.667	m(8) = 0.667	m(4) = 0.667	m(2) = 0.667	m(8) = 0.7	m(4) = 0.667	m(2) = 0.667	m(8) = 0.667	m(4) = 0.667
	m(3) = 0.333	m(9) = 0.333	m(5) = 0.333	m(3) = 0.333	m(9) = 0.3	m(5) = 0.333	m(3) = 0.333	m(9) = 0.333	m(5) = 0.333
3	m(1) = 0.667	m(10) = 0.667	m(3) = 0.667	m(1) = 0.667	m(10) = 0.667	m(3) = 0.667	m(1) = 0.667	m(10) = 0.667	m(3) = 0.667
	m(2) = 0.333	m(11) = 0.333	m(4) = 0.333	m(2) = 0.333	m(11) = 0.333	m(4) = 0.333	m(2) = 0.333	m(11) = 0.333	m(4) = 0.333
4	m(1) = 0.667	m(6) = 0.8	m(3) = 0.667	m(1) = 0.667	m(6) = 0.667	m(3) = 0.7	m(1) = 0.667	m(6) = 0.667	m(3) = 0.667
	m(2) = 0.333	m(7) = 0.2	m(4) = 0.333	m(2) = 0.333	m(7) = 0.333	m(2) = 0.3	m(2) = 0.333	m(7) = 0.333	m(4) = 0.333
5	m(1) = 0.667	m(3) = 0.667	m(2) = 0.5	m(1) = 0.667	m(3) = 0.667	m(1) = 0.7	m(1) = 0.667	m(3) = 0.6	m(1) = 0.667
	m(2) = 0.333	m(4) = 0.333	m(1) = 0.5	m(2) = 0.333	m(4) = 0.333	m(2) = 0.3	m(2) = 0.333	m(2) = 0.4	m(2) = 0.333
6	m(2) = 0.667	m(6) = 0.667	m(5) = 0.667	m(2) = 0.667	m(6) = 0.667	m(5) = 0.667	m(2) = 0.667	m(6) = 0.667	m(5) = 0.667
	m(3) = 0.333	m(7) = 0.333	m(6) = 0.333	m(3) = 0.333	m(7) = 0.333	m(6) = 0.333	m(3) = 0.333	m(7) = 0.333	m(6) = 0.333
7	m(1) = 0.667	m(7) = 0.667	m(3) = 0.667	m(1) = 0.667	m(7) = 0.667	m(3) = 0.667	m(1) = 0.667	m(7) = 0.667	m(3) = 0.667
	m(2) = 0.333	m(8) = 0.333	m(4) = 0.333	m(2) = 0.333	m(8) = 0.333	m(4) = 0.333	m(2) = 0.333	m(8) = 0.333	m(4) = 0.333
8	m(3) = 0.667	m(5) = 0.6	m(1) = 0.667	m(3) = 0.667	m(5) = 0.8	m(1) = 0.667	m(3) = 0.667	m(5) = 0.8	m(1) = 0.667
	m(4) = 0.333	m(6) = 0.4	m(2) = 0.333	m(4) = 0.333	m(6) = 0.2	m(2) = 0.333	m(4) = 0.333	m(7) = 0.2	m(2) = 0.333
9	m(2) = 0.9	m(10) = 0.6	m(4) = 0.667	m(2) = 0.75	m(10) = 0.9	m(4) = 0.667	m(2) = 0.8	m(10) = 0.9	m(4) = 0.667
	m(1) = 0.1	m(9) = 0.4	m(5) = 0.333	m(1) = 0.25	m(9) = 0.1	m(5) = 0.333	m(1) = 0.2	m(9) = 0.1	m(5) = 0.333
10	m(1) = 0.667	m(10) = 0.667	m(6) = 0.667	m(1) = 0.667	m(10) = 0.667	m(6) = 0.667	m(1) = 0.667	m(10) = 0.667	m(6) = 0.667
	m(2) = 0.333	m(11) = 0.333	m(7) = 0.333	m(2) = 0.333	m(11) = 0.333	m(7) = 0.333	m(2) = 0.333	m(11) = 0.333	m(7) = 0.333
11	m(1) = 0.667	m(10) = 0.667	m(5) = 0.667	m(1) = 0.667	m(10) = 0.667	m(5) = 0.667	m(1) = 0.667	m(10) = 0.667	m(5) = 0.667
	m(2) = 0.333	m(11) = 0.333	m(6) = 0.333	m(2) = 0.333	m(11) = 0.333	m(6) = 0.333	m(2) = 0.333	m(11) = 0.333	m(6) = 0.333
12	m(1) = 0.667	m(10) = 0.667	m(6) = 0.6	m(1) = 0.667	m(10) = 0.667	m(5) = 0.8	m(1) = 0.667	m(10) = 0.667	m(6) = 0.7
	m(2) = 0.333	m(11) = 0.333	m(5) = 0.4	m(2) = 0.333	m(11) = 0.333	m(4) = 0.2	m(2) = 0.333	m(11) = 0.333	m(5) = 0.3
13	m(1) = 0.667	m(10) = 0.667	m(5) = 0.8	m(1) = 0.667	m(10) = 0.667	m(5) = 0.667	m(1) = 0.667	m(10) = 0.667	m(5) = 0.667
	m(2) = 0.333	m(11) = 0.333	m(4) = 0.2	m(2) = 0.333	m(11) = 0.333	m(6) = 0.333	m(2) = 0.333	m(11) = 0.333	m(6) = 0.333
14	m(1) = 0.667	m(10) = 0.667	m(6) = 0.667	m(1) = 0.667	m(10) = 0.667	m(6) = 0.8	m(1) = 0.667	m(10) = 0.667	m(6) = 0.667
	m(2) = 0.333	m(11) = 0.333	m(7) = 0.333	m(2) = 0.333	m(11) = 0.333	m(7) = 0.2	m(2) = 0.333	m(11) = 0.333	m(7) = 0.333
15	m(2) = 0.667	m(7) = 0.95	m(3) = 0.667	m(2) = 0.667	m(7) = 0.667	m(3) = 0.667	m(2) = 0.667	m(7) = 0.667	m(3) = 0.7
	m(3) = 0.333	m(6) = 0.05	m(4) = 0.333	m(3) = 0.333	m(8) = 0.333	m(4) = 0.333	m(3) = 0.333	m(8) = 0.333	m(4) = 0.3
16	m(2) = 0.9	m(4) = 0.667	m(3) = 0.667	m(2) = 0.75	m(4) = 0.667	m(3) = 0.667	m(2) = 0.8	m(4) = 0.667	m(3) = 0.8
	m(1) = 0.1	m(5) = 0.333	m(4) = 0.333	m(1) = 0.25	m(5) = 0.333	m(4) = 0.333	m(1) = 0.2	m(5) = 0.333	m(2) = 0.2
17	m(2) = 0.667	m(5) = 0.9	m(3) = 0.667	m(2) = 0.667	m(5) = 0.9	m(3) = 0.667	m(2) = 0.667	m(5) = 0.6	m(3) = 0.667
	m(3) = 0.333	m(6) = 0.1	m(4) = 0.333	m(3) = 0.333	m(6) = 0.1	m(4) = 0.333	m(3) = 0.333	m(6) = 0.4	m(4) = 0.333

**Table 2:** Evaluation information by three experts.

Expert	BPA	
$E_{1}$	$m_{O1}^1$(3) = 0.4	$m_{O1}^1$(4) = 0.6
$E_{2}$	$m_{O2}^1$(3) = 0.9	$m_{O2}^1$(4) = 0.1
$E_{3}$	$m_{O3}^1$(3) = 0.8	$m_{O3}^1$(4) = 0.2

**Table 3:** The negation of BPAs.

Expert	BPA	
$E_{1}$	${\bar{m}}_{O1}^1$(3) = 0.6	${\bar{m}}_{O1}^1$(4) = 0.4
$E_{2}$	${\bar{m}}_{O2}^1$(3) = 0.1	${\bar{m}}_{O2}^1$(4) = 0.9
$E_{3}$	${\bar{m}}_{O3}^1$(3) = 0.2	${\bar{m}}_{O3}^1$(4) = 0.8

Then, we start to calculate the evidence distance between 3 experts. By using Eqs. (11) and (12), we get the evidence distance. Next we construct the distance matrix using Eq. (19):

$$\begin{aligned} {d} = \left( {\begin{array}{*{20}{c}} 0&{}{0.5}&{}{0.4}\\ {0.5}&{}0&{}{0.1}\\ {0.4}&{}{0.1}&{}0 \end{array}} \right) \end{aligned}$$

The smaller the distance between the two evidences, the higher their similarity. By using Eq. (20) we get the similarity matrix between 3 experts:

$$\begin{aligned} {Sim} = \left( {\begin{array}{*{20}{c}} 1&{}{0.5}&{}{0.6}\\ {0.5}&{}1&{}{0.9}\\ {0.6}&{}{0.9}&{}1 \end{array}} \right) \end{aligned}$$

The support degree reflects the degree of mutual support between evidence. We get the support degree by using the similarity matrix and Eq. (21), the results are shown in Table 4. Then we use the Eq. (22) to normalize the results to get the credibility, as shown in the Table 5. The more evidence is supported by other evidence, the more credible the evidence is.

**Table 4:** The support degree of evidence.

Expert	$E_{1}$	$E_{2}$	$E_{3}$
Support degree	1.1	1.4	1.5

**Table 5:** The credibility of evidence.

Expert	$E_{1}$	$E_{2}$	$E_{3}$
Credibility	0.275	0.350	0.375

Then, we use credibility as the weight of BPAs to calculate the ${\tilde{m}}_O^1$. The specific calculation process of $m_O^1(3)$ is shown as follows:

$$\begin{aligned} m_O^1(3)= & {} Crd(m_{O1}^1) \times m_{O1}^1(3) + Crd(m_{O2}^1) \times m_{O2}^1(3) + Crd(m_{O3}^1) \times m_{O3}^1(3)\\= & {} 0.275 \times 0.4 + 0.350 \times 0.9 + 0.375 \times 0.8\\= & {} 0.725 \end{aligned}$$

Next we use Murphy combination rule to get the final mass function, as shown in Table 6. Use Eq. (24) to get the average value of O risk factor. The specific calculation process is shown as follows:

$$\begin{aligned} O_{avg}^1= & {} 3 \times {\tilde{m}}_O^1(3) + 4 \times {\tilde{m}}_O^1(4)\\= & {} 3 \times 0.72 + 4 \times 0.28\\= & {} 3.12 \end{aligned}$$

In the same way, we can find out the average value of S and D risk factor. We put the results in Table 7.

Finally, we can get the improved RPN value:

$$\begin{aligned} RPN_{avg}^1 = O_{avg}^1 \times S_{avg}^1 \times D_{avg}^1 = 45.81. \end{aligned}$$

**Table 6:** BPAs based on Murphy combination rule.

BPA	Value
${\tilde{m}}_O^1(3)$	0.72
${\tilde{m}}_O^1(4)$	0.28

**Table 7:** The average value of 3 risk factors.

Risk factor	Value
$O_{avg}^{1}$	3.12
$S_{avg}^{1}$	7.25
$D_{avg}^{1}$	2.03

**Table 8:** RPN value based on different improved methods.

FMEA	AMWRPN^48^	MVRPN^60^	Improved MVRPN^30^	$RPN_{avg}$
FM1	5.1551	42.56	42.56	45.81
FM2	5.3171	64.00	64.05	70.27
FM3	3.8634	30.00	30.00	34.95
FM4	3.3302	18.00	17.97	20.61
FM5	1.6529	4.17	3.14	4.00
FM6	5.0964	60.00	60.00	65.94
FM7	3.3567	21.00	21.00	24.58
FM8	3.2975	15.00	15.00	17.42
FM9	8.3797	78.92	79.57	81.65
FM10	5.0964	60.00	60.00	68.66
FM11	4.7399	50.00	50.00	57.42
FM12	5.0973	50.00	50.00	63.13
FM13	4.9447	50.00	50.00	56.85
FM14	5.4187	60.00	60.04	68.13
FM15	5.9509	42.00	42.09	46.22
FM16	3.756	23.88	23.86	25.02
FM17	5.0554	30.05	30.05	32.92

## Discussion

**Figure 3:**
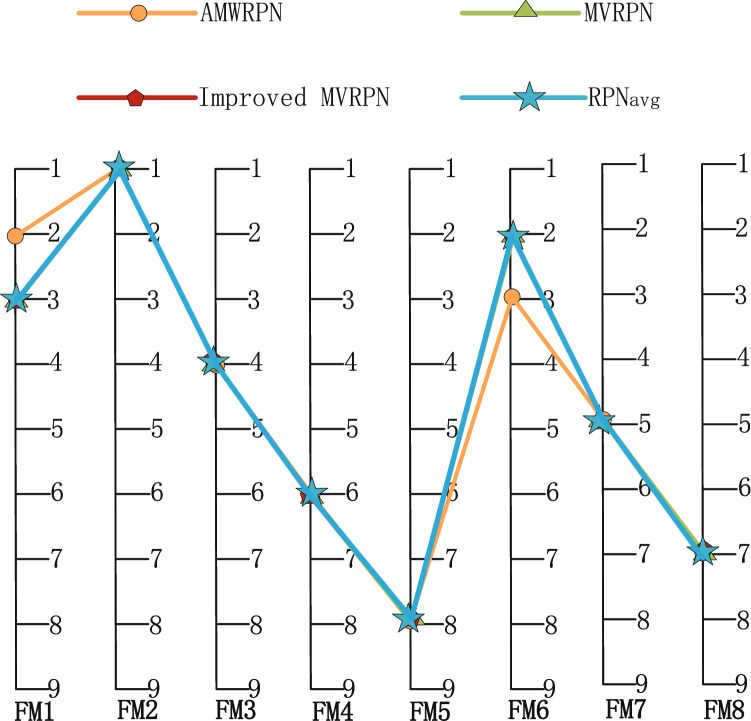
The ranking result of FM1–FM8.

**Figure 4:**
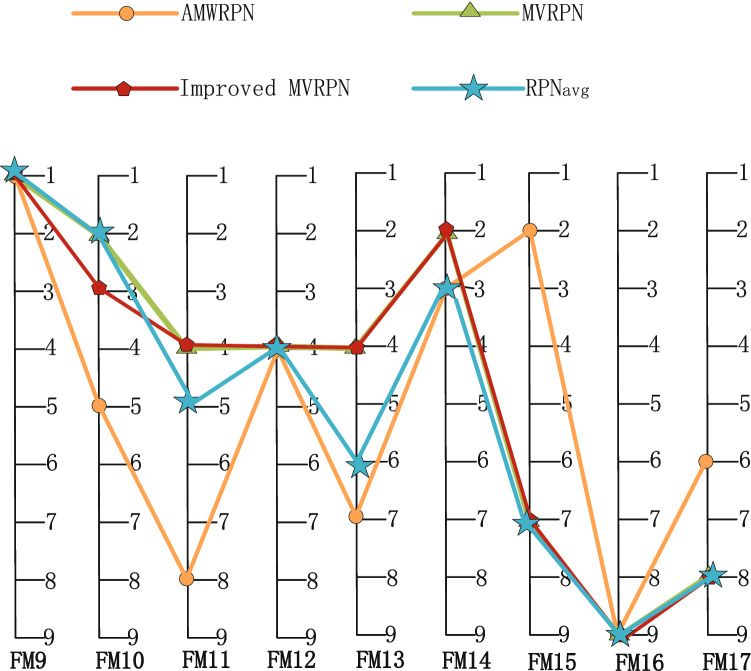
The ranking result of FM9–FM17.

After the same calculation process for 17 failure modes, using Eq. (25) we get the RPN value of each failure mode. The results ($RPN_{avg}$) are listed in the Table 8, as well as the results of some other methods. AMWRPN^48^ uses the method of Ambiguity Measurement to calculate the weight of experts. MVRPN^60^ averages the obtained RPN values. Improved MVRPN^30^ further improves the combination rule of MVRPN^60^. As described in the literature, $FM1 \thicksim FM8$ describe the failure mode of compressor rotor blades and $FM9 \thicksim FM17$ describe the failure mode of turbo rotor blades. Experimental results show that, for compressor rotor blades: $FM2\succ FM6\succ FM1\succ FM3\succ FM7\succ FM8\succ FM4\succ FM5$, for turbo rotor blades: $FM9 \succ FM10\succ FM14\succ FM12\succ FM11\succ FM13\succ FM15\succ FM17 \succ FM16$, $\succ$ indicates that the previous item has a higher priority. For compressor rotor blades and turbo rotor blades respectively, *FM*2 and *FM*9 have the highest priority, indicates that more resources should be allocated to it. In Fig. 3, we can know that our ranking result is nearly the same with others. In Fig. 4, we can find that the results calculated by different methods are slightly different, but this is acceptable. Because the calculation results of several groups in $FM9-FM17$ are very close, this brings some impact to our sorting results. The experimental results verify the rationality of our method.

## Conclusions

In the frame of D–S evidence theory, this paper proposes an improved failure mode and effects analysis method based on triangular fuzzy numbers, negation of BPAs and evidence distance. Firstly, the new BPAs was constructed by fuzzy modeling of expert evaluation results, then calculate the negation of BPAs, next BPAs weight was calculated by evidence distance, and finally, a new RPN value was calculated by fusion of Murphy combination rule. In short, the method considers how to fuse conflicting evidence from experts and also considers the relative weight inconsistency caused by the uncertainty of experts’ evaluation. We apply the proposed method to the failure mode analysis of compressor rotor blades and turbo rotor blades. The experimental results verify the effectiveness and rationality of this method. In the future research, we can broaden our approach to other fields. Moreover, we can consider the relative importance of each risk factor to enrich our study.

## Data Availability

All data generated or analysed during this study are included in this published article.
